# A novel *Bacillus pumilus*-related strain from tropical landfarm soil is capable of rapid dibenzothiophene degradation and biodesulfurization

**DOI:** 10.1186/s12866-014-0257-8

**Published:** 2014-10-08

**Authors:** Elizandra Bruschi Buzanello, Rachel Passos Rezende, Fernanda Maria Oliveira Sousa, Eric de Lima Silva Marques, Leandro Lopes Loguercio

**Affiliations:** Department of Biological Sciences, State University of Santa Cruz – UESC, Rod. BR 415, Km 16, 45662-900 Ilhéus, BA Brazil; Faculty São Miguel do Iguaçu – UNIGUAÇU/FAESI, Rua Valentin Celeste Palavro, 1501, Jardim Panorama, 85877-000 São Miguel do Iguaçu, PR Brazil

**Keywords:** *B. pumilus*, Dibenzothiophene sulfoxide, 2-hydroxybiphenyl-2-sulfinate, 4S pathway, Bioremediation

## Abstract

**Background:**

The presence of organic sulfur-containing compounds in the environment is harmful to animals and human health. The combustion of these compounds in fossil fuels tends to release sulfur dioxide in the atmosphere, which leads to acid rain, corrosion, damage to crops, and an array of other problems. The process of biodesulfurization rationally exploits the ability of certain microorganisms in the removal of sulfur prior to fuel burning, without loss of calorific value. In this sense, we hypothesized that bacterial isolates from tropical landfarm soils can demonstrate the ability to degrade dibenzothiophene (DBT), the major sulfur-containing compound present in fuels.

**Results:**

Nine bacterial isolates previously obtained from a tropical landfarm soil were tested for their ability to degrade dibenzothiophene (DBT). An isolate labeled as RR-3 has shown the best performance and was further characterized in the present study. Based on physiological aspects and 16 s rDNA sequencing, this isolate was found to be very closely related to the *Bacillus pumillus* species. During its growth, high levels of DBT were removed in the first 24 hours, and a rapid DBT degradation within the first hour of incubation was observed when resting cells were used. Detection of 2-hydroxybiphenyl (HBP), a marker for the 4S pathway, suggests this strain has metabolical capability for DBT desulfurization. The presence of MgSO_4_ in growth medium as an additional sulfur source has interfered with DBT degradation.

**Conclusions:**

To our knowledge, this is the first study showing that a *Bacillus* strain can metabolize DBT via the 4S pathway. However, further evidences suggest RR-3 can also use DBT (and/or its derivative metabolites) as carbon/sulfur source through another type of metabolism. Compared to other reported DBT-degrading strains, the RR-3 isolate showed the highest capacity for DBT degradation ever described in quantitative terms.

The potential application of this isolate for the biodesulfurization of this sulfur-containing compound in fuels prior to combustion was discussed.

## Background

Sulfur-containing organic compounds are present as a substantial portion of petroleum, and generally display significant economic and environmental impacts. Among these, health issues (e.g. mucosal irritation and muscle and bronchial spasms), corrosion of equipment in refineries, acid rain [1,2], damage of crops (when present in the soils), reduction of the pH of lakes, and increased damage to marine life [3] can be highlighted. Because those compounds can be air transported, their impact tends to be also observed at a distance from the sites of production and/or use. However, all these damages can be sharply reduced if sulfur-rich compounds are removed from petroleum and its derivatives before they go through combustion [4]. In oil refineries, various hydrodesulfurization processes are usually adopted for sulfur removal [5], although the temperature and pressure management costs are usually very high and the removal process is difficult and largely inefficient [6]. In this scenario, biological desulfurization is an important alternative to oil industries, as it takes place under mild conditions of temperature and energy [7]. Efficient biodesulfurization of sulfur-containing compounds is the most economically and environmentally friendly manner to eliminate this class of important pollutants from the production/consumption chains of fossil fuels.

Studies on biodesulfurization have considered dibenzothiophene (DBT) as a model compound [8-10]. Desulfurization of this molecule is essentially achieved through either reductive or oxidative pathways that release sulfur in the form of sulfate or sulfite [10-13]. A biodesulfurization process known as the ‘4S’ pathway is of particular interest, as it allows the maintenance of a large calorific value of fuels, with less potential for environmental pollution; it was discovered after a series of studies involving the *Rhodococcus* sp. isolate IGTS8 [14,15]. In this pathway, a specific oxidative attack against the thiophenic compound causes removal of the sulfur molecule. Briefly, this pathway displays various enzymes with different specific activities [3,14], which act in the following sequence of steps [15]: a monooxygenase (the ‘DszC’) oxidizes DBT into DBT sulfoxide (DBTO), another converts this compound into DBT sulfone (DBTO_2_), which is then converted into 2-hydroxybiphenyl-2-sulfinate (HBPS); as the last step, a desulfinase (the ‘DszB’) withdraws sulfur from the carbon-sulfur bonds, transforming HBPS into 2-hydroxybiphenyl (HBP) and sulphate. Bacteria that use this metabolic pathway can, therefore, remove the highly toxic thiophenic compound in the form of a less aggressive inorganic substance, in a biochemical process that prevents losses of energy value [9,11]. Although some authors have added extra energy sources in growth media in an attempt to increase the degradation capability, the use of specialized microorganisms for the biodesulfurization procedure usually does not require media enrichment, such that the test compound (e.g. DBT) can be used as the sole source of energy for the microbial metabolism [16-18].

In a previous study, the use of DBT as an energy source was assessed in the growth of bacteria obtained from soil from an oily sludge landfarming in a tropical environment [19]; nine isolates that showed DBT-consuming ability were selected for further research. In the current work, these isolates were compared in their growth behavior in media containing DBT and were identified through sequencing. The isolate labeled as ‘RR3’ showed the best DBT-degrading performance and was further assessed, aiming at verifying its temporal and quantitative ability to not only degrade this compound, but also to produce HBP, one of the final products of the 4S pathway resulting from the DszB enzyme activity. The results indicated this isolate as being closely related to *Bacillus pumilus* and as being capable of a very high and rapid degradation of DBT, likely through more than one type of metabolism.

## Results and discussion

All the nine isolates under study (labeled from an ‘RR’ series as RR-3, −14B, −19, −31B, −33b, −43, −52, −25A and -33o) showed to be capable of biodesulfurization of oils, as they were able to grow in minimal mineral medium supplemented with DBT as the sole sulfur and carbon source. Their molecular identification was carried out by sequencing of the *16 s rRNA* gene, obtained by PCR amplification and direct amplicon purification from each isolate. As shown in Table 1, all strains appeared to belong to the phylum Firmicutes, Bacilli class and Bacillales order. At the genus level, only the -33o isolate was classified as *Paenibacillus* sp (Paenibacillaceae), whereas all others appeared to belong to the *Bacillus* genus (Bacillaceae). Further support to the assignment of these isolates to the *Bacillus* genus was given by positive results for the Gram test, as well as by cells morphology under light microscopy. Seven out of the nine isolates could not be resolved between *Bacillus safensis* and *B. pumilus* based only on the sequence of this gene (Table 1). These two closely related species have a phylogenetic relationship that has already been fully described [20].

**Table 1:** **Taxonomic identification of nine DBT-consuming bacterial isolates through 16S rRNA gene sequencing**

**Isolates**	**Size (bp)** ^***1***^	**Access numbers** ^***2***^	**Identity (%)** ^***3***^	**Max score**	**Taxon**
−3,-14B,-19, −31B,-33b, −43,-52	592-598	NR_074977	99	1092	*Bacillus pumilus*
		NR_041794			*B. safensis*
−25A	598	NR_027552	100	1105	*B. subtilis*
−33o	578	NR_040884	96	955	*Paenibacillus illinoisensis*

^*1*^Length of PCR-amplified fragment that produced good quality sequences.

^*2*^Sequences deposited in the GenBank that gave the best alignment results from BLAST search.

^*3*^‘e-values’ were all equal to zero and query coverages were all 100%.

Nevertheless, taking into account other studies on morphological, physiological and PCR amplification aspects of this RR-3 isolate (data not shown) and the other six closely related ones (Table 1), we strongly suggest these seven isolates belong to the same species, and that they are indeed more closely related to the *B. pumilus* species. Interestingly, with exception of the *Paenibacillus* sp. isolate identified, this appears to be the first report in which isolates/species from the *Bacillus* genus have shown ability to grow on/metabolize DBT (Table 2). This is somewhat not surprising, as *Bacillus* spp is one of the most studied bacterial genus, showing a great metabolic versatility that provides the opportunity for an array of biotechnological applications [21]. Adding strength to this possibility, a recent genome sequencing of the *B. pumilus* S-1 strain indicated the presence of various monooxygenases [22], which could help explain at least part of the observed DBT-desulfurization phenotype of RR-3. Moreover, such genome characterization also found an array of other metabolical/biochemical capabilities, suggesting ability of this species in using a variety of carbon sources and adapting to various unfavorable conditions [22]. However, in our hands, the use of specific PCR amplification for the *dszC* and *dszB* genes (of the 4S pathway) resulted in non-specific amplicons (characterized by sequencing). Moreover, sequence alignments of *dsz* genes (*A*, *B* and *C*) for the *Rhodococcus* sp and *Gordonia alkalivorans* bacterial species (accession numbers L37363 and EU364831 publicly available) against the full genome of *B. pumilus* [22] have not provided any meaningful result (data not shown). Taken together, these results suggest the RR-3 isolate is likely bearing a specific metabolism and/or novel undescribed genes for DBT degradation that has not yet been described (see more discussion below).

**Table 2:** **DBT degradation by different bacterial strains**

**DBT degrad. (%)**	**Time** ^***1***^ **(h)**	**Strains**	**Reference**
43	24	*Sphingomonas* sp.	Gai, et al. [23]
82	48	*Stenotrophomonas* sp. NISOC-04	Papizadeh et al. [6]
50	120	*Microbacterium* strain ZD-M2	Li et al. [24]
58	80	*Alcaligenes denitrificans (*subsp*)*	Van Afferden et al. [16]
100	75	*Rhodococcus erythropolis* SHT87	Davoodi-Dehaghani et al. [25]
60	120	*Shewanella putrefaciens* NCIMB 8768	Ansari et al. [26]
83	120	*Rhodococcus erythropolis* IGTS8	Ansari et al. [26]
75	36	*Rhodosporidium toruloides* DBVPG 6662^*2*^	Baldi, et al. [27]
*99.9*	*24*	*Bacillus* sp. RR-3	*This study*

^*1*^Time of culturing/incubation in which the corresponding % of DBT degradation on the left column was obtained.

^*2*^An yeast species.

These results thereby suggest that landfarm soil (at least in tropical environments) may be a rich source of other bacterial species not yet identified or recognized as efficient DBT degraders.

With the objective of verifying the best growth performances in medium containing dibenzothiophene as the sole carbon source (minimal medium + 0.5 mM DBT), all nine isolates were cultivated in these same conditions. The results indicated that the *B. pumilus*-related RR-3 isolate showed the fastest growth behavior among all, which clearly suggests an intrinsic and special capability of using DBT as an energy source compound. Based on this, we opted for pursuing further in-depth characterization only for this isolate, which was considered a representative of the group with the six other very similar *B. pumilus*-like isolates (Table 1).

**Figure 1:**
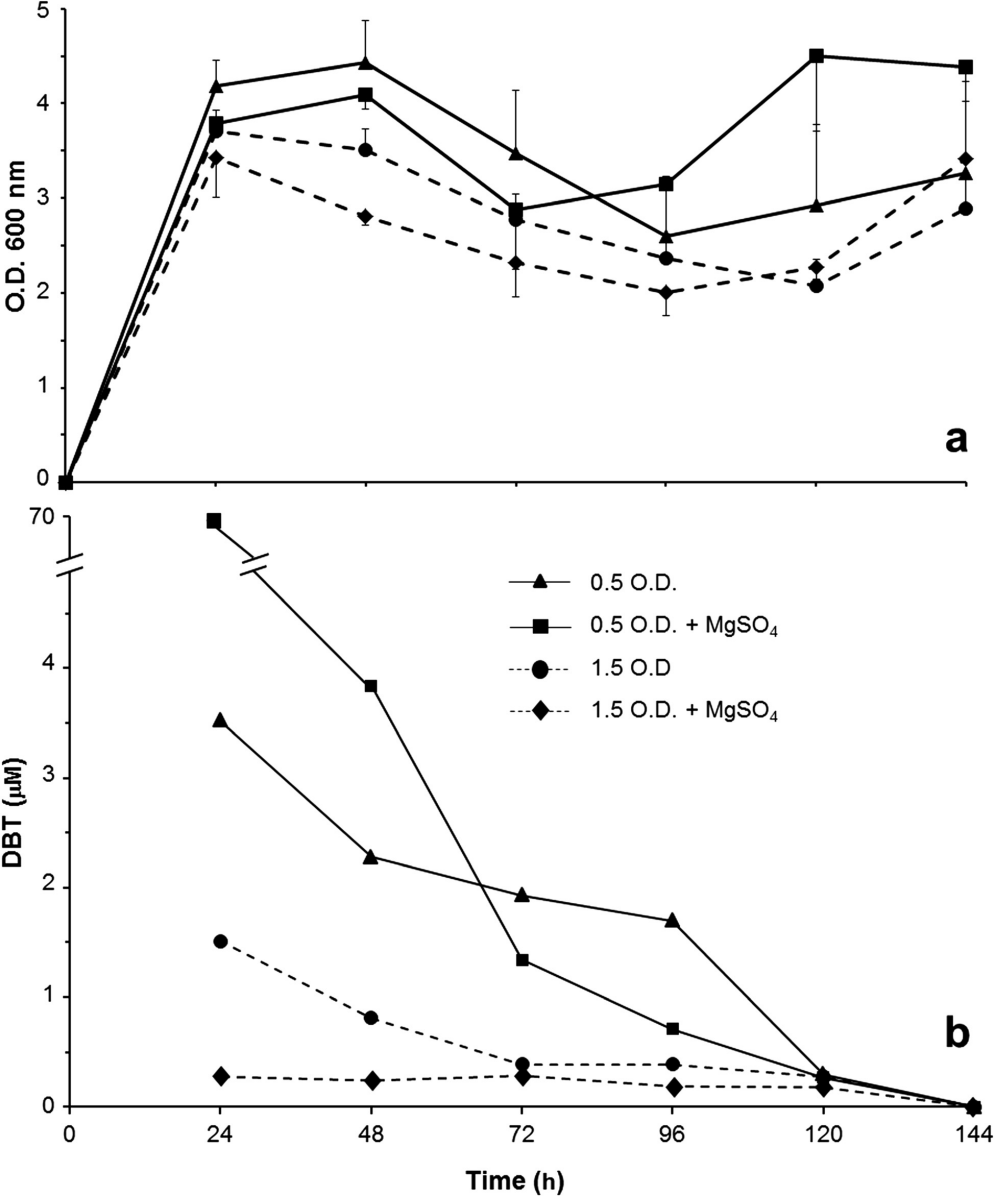
**Temporal profiles of** ***Bacillus pumilus*** **-like RR-3 strain in LB + 0.5 mM DBT media. (a)** Growth curve assessed by optical density (OD at 600 nm) of cell mass in culture at different times. **(b)** Residual DBT concentration (μM) in culture media at the same time points. As indicated by the legend in the graph, the four different growing conditions were established in 10-mL cultures by varying the presence of MgSO_4_ (6.65 μM) in the medium, combining with two amounts of inoculum cells. The RR-3 inocula were established by adding pre-cultured cell suspensions, with 0.500 and 1.500 OD readings at 600 nm, at 5% of final culture volume. Treatments were applied in triplicate and the experiment was repeated at least once, with same results. Hexane extraction and HPLC analysis were used for detection of both compounds. Practically the whole amount of the applied DBT could be detected at time zero.

The RR-3 isolate was further characterized with regards to not only aspects of its physiology when growing in DBT-containing LB media, but also the formation of HBP, one of the end products in the 4S biodesulfurization pathway [15]. Firstly, we evaluated the kinetics of growth and DBT consumption in LB culture based on optical densities, assessing these variables in relation to presence of MgSO_4_ (an alternative sulfur source) and different amounts of inoculum cells (Figure 1). When these cells originated from a pre-inoculum with an OD of 0.5 at 600 nm, the RR-3 isolate entered the exponential phase at around 12 h of growth (not shown), and the stationary phase between 30 and 48 h. A different temporal pattern was noticed when the inoculum had ~3 × more cells: after 24 h of growth, a decline in OD values was observed, which went up again only after 120 h, whereas for 0.5-OD treatments (less inoculum), such a second growth stage occurred between 72 and 96 h (Figure 1a). Parallel to this, a high DBT consumption was observed since early stages of growth. Considering the initial concentration of 500 μM of DBT in the medium for both inoculum amounts, it was striking that this compound was almost completely consumed (>99%) after the first 24 h (1 – 4 μM DBT left in the medium), for both 0.5- and 1.5-OD inocula (Figure 1b), despite of their distinct growth profiles (Figure 1a). From the 48-h time point forward, the very little remaining DBT appeared to be used up, taking longer for that to occur when inoculum was less concentrated (Figure 1b). The possibility of DBT being spontaneously degraded in such a fast rate in LB medium under the same conditions of culture can be discarded. Experiments with *Arthrobacter* sp. have shown strong evidence for its stability, as it remained unreacted after two weeks in a medium with boiled bacteria [28]. This was indeed confirmed experimentally in our case, based on a full recovery of applied DBT (~500 μM) in microbe-free LB medium, after incubation for the same time and culture conditions.

After more than 72 h of culture (Figure 1a), the observed OD patterns suggest a diauxy behavior for the RR-3 strain, as it seemed to have entered a second growth phase. As observed by Li et al. [29] with phenol degradation such a behavior occurs when more than one energy source is, or become available in the medium. Although the activation of many genes for certain metabolisms is directly related to cell density (i.e. *quorum sensing* systems) [30,31], the remaining amounts of DBT after 24 h of growth for both inoculum concentrations (pre-inocula at 0.5 and 1.5 OD – Figure 1b) did not allow us to verify whether this possibility was occurring with regards to sensing/signaling in relation to DBT. Nevertheless, due to the fast consumption of DBT early in culture, it is reasonable to assume that other carbon sources, either directly from the medium itself or as a consequence of culture metabolism, have likely been used for the diauxy behavior to occur. In this case, we cannot rule out the possibility of *quorum sensing* systems being operational at those later stages in culture, although an exact mechanism cannot be proposed at this time.

The cells of the *B. pumilus*-like RR-3 isolate seemed to adhere to DBT crystals, as observed at 24-h culture (data not shown). A similar behavior was observed for yeasts [27], which appears to be a strategy to reduce the cell-compound interface to facilitate enzymatic attack, despite some morphological changes that have occurred as a consequence. No cell abnormality was noted in the RR-3 isolate when attached to DBT crystals (at 1000 × magnification), although this requires further in-depth observations for more conclusive results. Nevertheless, this physical attachment of RR-3 cells to DBT crystals suggest they can interact directly with this compound, and so, favored its metabolization, which may occur independently of being cultured/incubated in richer or simple media (see discussion on resting cells assays further below).

Addition of very little amounts of an alternative sulfur source (~7 μM MgSO_4_) in the medium containing DBT did not lead to any clear modification in the OD values for cell mass, considering both inoculum amounts (Figure 1a). However, with regards to the DBT remaining in culture, an interesting alteration was observed: whereas 20 × more DBT was observed for 0.5-OD inoculum in medium containing MgSO_4_, essentially no DBT was found in the same medium when culture started from the 1.5-OD inoculum (Figure 1b). These results are suggesting that a disturbing effect of MgSO_4_ in the metabolism of DBT by the RR-3 isolate may be observable, depending on the combination of inoculum amount and MgSO_4_ concentration. A similar interference on degradation pathways of organic compounds by readily available inorganic sulfur sources have also been reported in other systems [5,32]. At a higher inoculum concentration (cell density), no DBT left for consumption was found (Figure 1b); such a condition may not be unexpected, as it depends on the dynamics of DBT degradation, the initial amount of MgSO_4_ used, and the culturing time allowed prior to the evaluation. Considering the potential use of this *B. pumilus*-like isolate for either DBT biodesulfurization from fuels, or its removal from the environment in bioremediation processes, our data indicated that inorganic sulfur sources, if present in sufficient amounts, can interfere with these biodesulfurization processes. At this point, we can not state whether such interference is only of an inhibitory nature; further investigation on combining different concentrations of MgSO_4_ and inocula, observing these effects in earlier culture times is required to sort this out.

**Figure 2:**
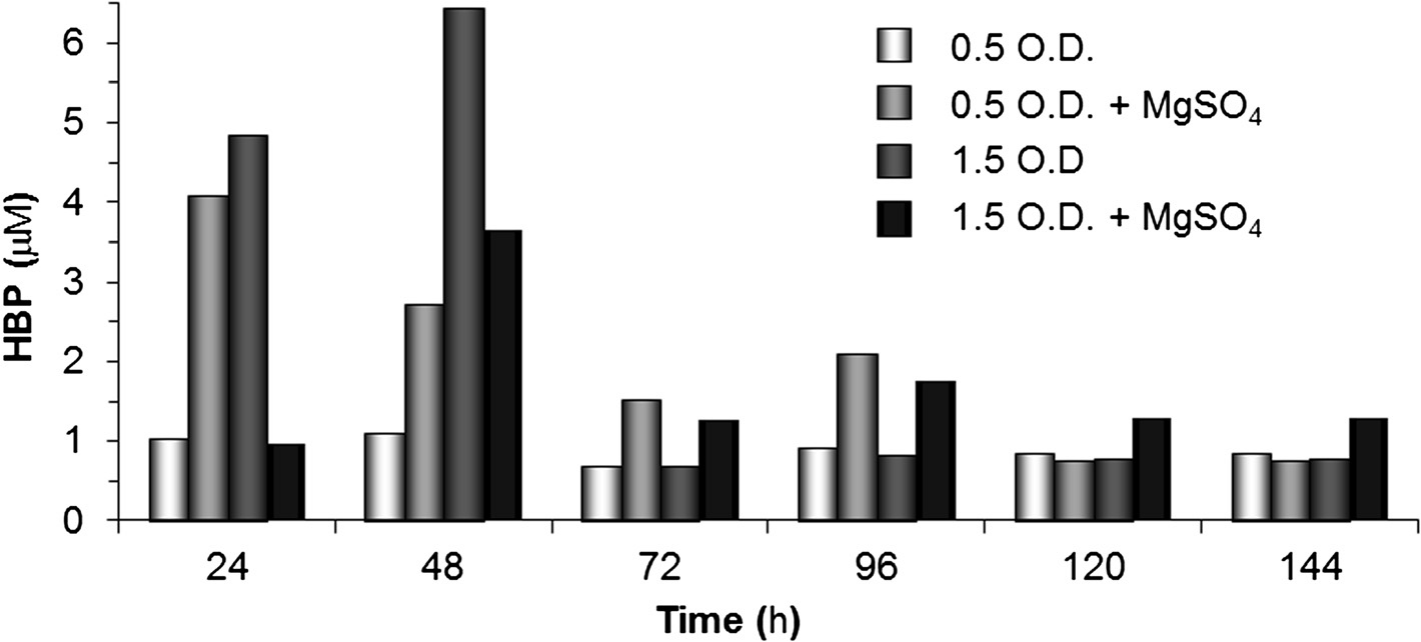
**Detection of 2-hydroxybiphenyl (HBP) in culture of** ***B*** **.** ***pumilus*** **-like RR-3 strain in LB + 0.5 mM DBT media.** HBP levels were gauged by hexane extraction and HPLC analysis. Media treatments, experimental design and culture times evaluated are the same described in Figure 1.

Detection of end products of a given pathway is an indication that such metabolism might well be operational. Hence, we assessed the presence of 2-hydroxybiphenyl (HBP) in the four media under test to verify whether the RR-3 *Bacillus pumilus* isolate is able to desulfurize DBT through a pathway related to the ‘4S’. HBP levels ranging from 0.5 to 6.5 μM were detected for all cultures and throughout all times, although > 2.5 μM HBP was observed only up to 48 h (Figure 2). However, a detailed analysis of the results did not allow us to recognize any pattern of HBP presence/accumulation that could be associated with the patterns of DBT degradation. For instance, the high levels of DBT remaining in the 0.5-OD + MgSO_4_ medium (Figure 1b) did not correspond neither to highest nor to lowest HBP levels (Figure 2); similarly, the highest levels of HBP at 24 and 48 h of culture in 1.5-OD medium did not correspond to either high or low levels of remaining DBT (Figures 1b and 2). Moreover, variation in cell density given by different inocula did not specifically correlate with higher or lower levels of HBP, at least under these experimental conditions.

By comparing the overall low levels of remaining DBT after only 24 h in culture with the ~100 times higher starting level of DBT in the media (500 μM), it seems clear that a fast-consumption metabolism is occurring. In this circumstance, it would be reasonable to expect that stoichiometric high levels of HBP would be found in culture from the use of DBT at the beginning of the 4S pathway. However, the relatively low overall levels (<7 μM) of HBP detected and the lack of a pattern for its accumulation in the tested media (Figure 2) suggest that most of the HBP produced may have been somehow consumed or degraded after its synthesis. Alternatively, DBT and/or its derivative metabolites might have been used as carbon source in an alternative pathway [16-18], thereby preventing full operation of the 4S pathway, and so, the production/accumulation of HBP. At least as observed for the culture starting with 0.5-OD inoculum, the rapid DBT consumption and detection of HBP could corroborate previous findings about the action of 4S-pathway enzymes during exponential growth phase [6,23-26,33,34]. Although the HBP detection data would also indicate that genes related to this metabolism (such as *dsz*C and *dsz*B) should be operational, homologous sequences for these genes were lacking (see above), which suggest the existence of a specific mechanism and genes not yet described for DBT consumption/HBP formation in the *B. pumilus*-like RR-3 isolate. To account for the lack of stoichiometric relationship between DBT and HBP, the existence of another DBT-consumption metabolism that includes the destruction of the molecule ring cannot be discarded (see below). Further research involving biochemical/molecular tests and full-genome sequencing is currently underway to address this question in more details.

To our knowledge, the data presented thus far indicated this strain seems to be the fastest and most efficient degrader of DBT already described, at least at such an initial concentration of this compound in the culture media. From a series of recent studies dealing specifically with DBT-degrader microbes (Table 2), the closest efficiency in this process in relation to RR-3 was found for *Stenotrophomonas* sp., which showed 82% consumption of initial DBT, after 48 h in culture; *Rhodococcus erythropolis* consumed all the DBT applied, but it took ~3 × longer to achieve this. Considering the same 24-h time in culture for the RR-3, *Sphingomonas* sp showed to degrade only 43% of initial DBT (Table 2). It is worth mentioning that our working concentration for DBT in the media fell within the range of initial amounts tested in degradation studies by other microorganisms, which varied from 0.2 – 0.25 mM [25] up to 0.8 – 1.0 mM DBT [6]. In a report where the DBT degradation ability of *Shewanella putrefaciens* and *Rhodococcus erythropolis* were studied, normal growth occurred at initial DBT levels of 0.3 mM, but inhibitory effects were observed at concentrations ≥ 0.6 mM [26].

**Figure 3:**
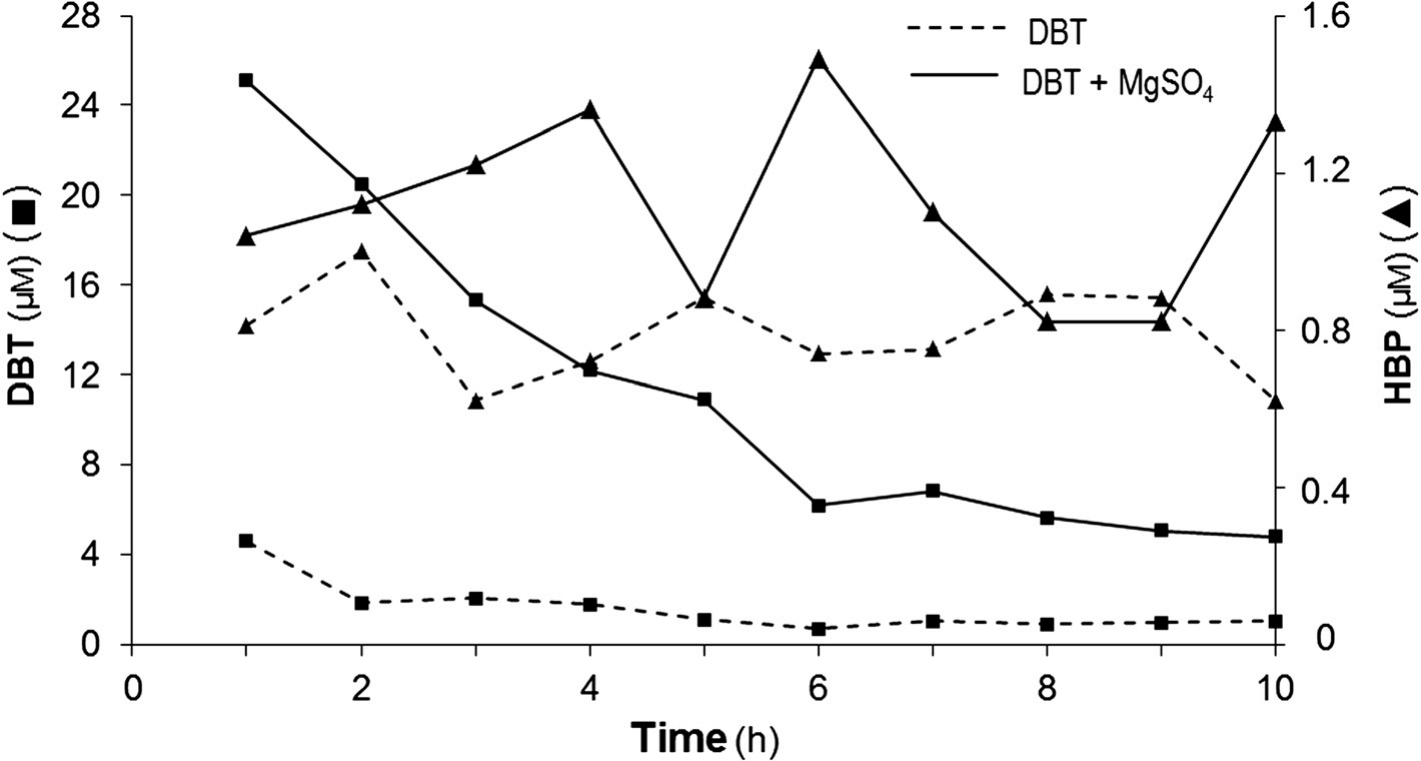
**Kinetics of DBT consumption and HBP detection in** ***B*** **.** ***pumilus*** **-like RR-3 strain in resting cells assays.** Dashed-line curves correspond to phosphate buffer + 0.5 mM DBT and solid-line curves to this medium also containing 6.65 μM MgSO_4_ as additional sulfur source. The inoculum of the strain was done as described, with cell amounts corresponding to a pre-inoculum suspension with a 0.500 reading of OD at 600 nm. Vertical axis on the left (■) indicate residual concentrations of DBT in the cell suspension and on the right (▲), the detected levels of HBP. Treatments were applied in triplicate. Detection method and levels of retrieval of the applied DBT were the same indicated in the legend of Figure 1.

Considering the fast DBT consumption / HPB detection observed during RR-3 growth, a further assay involving resting cells of this isolate was performed, aiming at assessing the rate of DBT degradation and possible presence of HBP in the first 10 h of exposure to DBT (Figure 3). It is worth noting that the cells in this assay were collected from a culture in logarithmic phase, with their physiology presumably set at an active metabolic state. The results confirmed a very fast consumption of DBT by this strain, with an astonishing degradation rate of ≥ 95% of initial DBT after only the first hour of incubation, depending on whether MgSO_4_ is present or not. Again, the presence of this alternative sulfur source delayed the use of DBT by the bacterial cells, leaving ~5 × more of residual DBT in the first hour and inducing its full consumption to take longer than 10 h (Figure 3). This finding adds further strength to the suggested interference of inorganic sulfur onto DBT metabolism by the RR-3 strain (Figure 1b; [5]). It is critical to note that this compound was present as the sole carbon source available for the cells in simple phosphate buffer. As discussed above, DBT is a very stable component, so that one should not expect any decrease in its amount only by incubation in this buffer at such a mild temperature of 30°C and a slow speed of 150-rpm shaking. Therefore, such a very rapid disappearance of DBT can only be attributed to a very fast metabolization by the resting cells. Since the very low levels of HBP detected at the same time were not stoichiometric compatible to such a large DBT degradation, this, in fact, adds support to the possibility of a concomitant existence of an alternative metabolism for energy production (directly and/or through derivative/intermediate metabolites) using DBT as carbon source. Further experiments to specifically address this question of more than one DBT-consumption pathways in RR-3 isolate are certainly warranted to clarify this issue.

Taking into account that *(i)* the cells were harvested when they were growing exponentially, with oxidative enzymes and their genes being expectedly active, for the resting-cells assays, *(ii)* DBT was readily consumed when present as the single carbon source, and *(iii)* in nutrient-rich medium (such as LB), DBT was yet consumed during the first stages of growth in cultures that reached stationary phase and diauxy behavior later than 24 h (Figure 1a), it is strongly suggested that DBT consumption (as energy source and/or in a desulfurization pathway) by the *B. pumilus*-like RR-3 strain is metabolically preferential, when considering other carbon sources available. A high adaptation ability of this bacterium to the surrounding conditions provided by the landfarm system is a plausible explanation for this phenomenon. With regards to HBP, it was once again detected in the cell suspension at very low concentrations (0.6 – 1.6 μM range), with no observable accumulation pattern in relation to DBT consumption (Figure 3). Hence, based on the above discussion, HBP is likely being produced as a consequence of DBT metabolism through a 4S-like (but not equal to) pathway, but used up by the resting cells and/or not being synthesized at all, assuming in this case a concomitant DBT consumption by a different energy-production metabolism, as mentioned above. Although HBP was found within a small concentration range, a higher oscillating behavior shown in Figure 3 for HBP presence was observed for the MgSO_4_-containing treatment, likely because there was DBT still left to be metabolized in the medium.

## Conclusions

From this study, we concluded that *(i)* landfarm soil from tropical regions provides a rich source of alternative microbes and metabolisms for biodesulfurization processes; *(ii)* this seems to be the first report of a bacterium from the *Bacillus* genus (most closely related to *B. pumilus*) as having such a great ability in metabolizing DBT; *(iii)* this *B. pumilus*-like RR-3 strain showed the greatest degrading potential ever described for other bacterial strains, based on growth kinetics in liquid culture and resting cells assays; *(iv)* evidence for the preferential use of DBT as carbon source, likely in more than one type of degrading metabolism, was shown by the lack of a pattern or stoichiometric relationship between DBT consumptioin and HBP detection. Microorganisms displaying a biodesulfurization pathway in its metabolic framework similar to the ‘4-step’ one have great biotechnological potential for DBT removal in fossil fuels production; they also have environmental significance in bioremediation strategies for polluted areas, through direct DBT consumption/degradation via other types of metabolism. Further assessment of other intermediate metabolites known to be produced by other DBT desulfurization pathways in culture are required to shed more light on the biotechnological potential of the *Bacillus pumilus*-like RR-3 strain.

## Methods

### Microorganisms

The bacterial strains used in this study were isolated by Maciel et al. [19] from landfarm soil of the Landulpho Alves refinery (Bahia, Brazil). They were labeled as RR-3, RR-14B, RR-19, RR-31B, RR-33b, RR-43, RR-52, RR-25A and RR-33o. The strains were inoculated in triplicate in test tubes with solid LB medium, and incubated for 24 hours at 30°C. After growth, sterile mineral oil was added to the tubes and strains were stored either in a refrigerator at 4°C or in freezer at −20°C, in Nuc tubes containing liquid LB + glycerol at 10% final concentration.

### Bacterial DNA isolation, amplification and sequencing

The strains were inoculated into 2-mL microfuge tubes containing 1 mL of TY medium (5.0 g tryptone, 3.0 g yeast extract, 0.9 g dehydrated calcium chloride, 15.0 g agar-agar per liter of doubled-distilled water). The microtubes were incubated under constant shaking of 150 rpm, at 30°C for 48 h. After growth, the cultures were centrifuged for 7 min at 13 krpm and the supernatant was discarded. Total DNA was extracted as described [35].

Extracted DNA was further PCR-amplified with the 16 s rDNA universal primers F27 (5′-AGAGTTTGATCMTGGCTCAG-3′) and R1525 (5′-AAGGAGGTGWTCCARCC-3′) [36] in 25-μL reactions containing 3 mM MgCl_2_, 1× PCR buffer, 0.2 mM each dNTP, 5 pmol each primer, 0.05 U μL^−1^ Taq DNA polymerase (Promega®) and 1.5 μL DNA. Thermal cycler was programmed for amplification as follows: an initial denaturation step at 94°C for 5 min, followed by 35 cycles of 1-min denaturation at 94°C, 1-min annealing at 57°C, and 3-min extension at 72°C, with a final extension step at 72°C for 15 min. Amplification was checked by electrophoresis in 1% agarose gel, with amplicons visualized through UV light after ethidium-bromide staining. DNA purification from PCR was done with 3 M sodium acetate and absolute isopropanol on a regular precipitation-washing scheme, being further quantified by spectrophotometry. Samples were prepared as specified by the sequencing-service provider (Ludwig Biotec Inc., Porto Alegre-RS, Brazil): microtubes containing 60 ng of purified PCR product and 4.5 pmol of the sequencing primer F27 (5′-AGAGTTTGATCMTGGCTCAG-3′) were dried in an Eppendorf 5301 ‘speed-vac’ concentrator and sent out for sequencing (procedures are available by the service provider upon request). The electropherograms generated were analyzed by the Phred/Phrap/Consed software suites to remove low-quality sequences [37]. After processing, the nucleotide sequences were compared with those at the GenBank database, using the Basic Local Alignment Search Tool (BLAST) [38]. The softwares used for sequences alignment and dendrogram development were the ‘ClustalW’ and ‘MEGA5’ [39,40], respectively. The best sequence match obtained per isolate to provide the closest taxonomic identification was based on the highest identity score, coupled with the highest size of query fragment covered in the alignment.

### Growth performance of isolates in DBT

Growth behaviors in DBT were evaluated for the previously selected nine bacterial isolates, which were subjected to a two-step culturing procedure [41], based on optical density (OD) of cells, although with a modified protocol. First, a loopful of bacterial cells from stocks of each isolate was inoculated in a Petri dish containing solid LB-medium supplemented with 0.5 mM DBT. These plates were incubated for 24 h at 30°C. After growth of the isolates, a ‘pre-inoculum’ suspension was prepared by adding bacterial cells (scraped from the Petri dishes) in a sterile 0.85% saline solution, up to a final concentration corresponding to an OD of 0.500 at 600 nm. In a second culturing step, 10-mL cultures per isolate were set by adding aliquots of 0.5 mL of the pre-inoculum suspensions to 125-mL flasks containing 9.5 mL of mineral minimal medium; this medium was prepared by adding 0.5 g KH_2_PO_4_, 4.0 g K_2_HPO_4_, 1.0 g NH_4_Cl, 0.02 g CaCl_2_, 0.01 g NaCl and 0.2 g MgCl_2_, plus 10 mL of trace elements solution (containing 0.5 g/L FeCl_3_, 0.5 g/L ZnCl_2_, 0.5 g/L MnCl_2_, 0.1 g/L Na_2_MoO_4_, 0.05 g/L CuCl_2_ and 0.05 g/L Na_2_WO_4_), all being dissolved directly in a liter of doubled-distilled water prior to autoclaving. DBT was added to this autoclaved medium at a 0.5 mM final concentration as the sole carbon-sulfur source. The flasks were incubated at 150 rpm on a rotatory bench shaker, at room temperature for 72 h. The experiment was performed in triplicate.

### Growth kinetics of the selected strain

The temporal growth pattern of the selected RR-3 isolate was determined by OD measurements at 600 nm, with bacterial growth being assessed every 24 hours, up to 144 hours (time ‘zero’ was read right after inoculation). Different growth treatments were established by varying the presence of MgSO_4_ in DBT-containing LB medium, and the inoculum concentration, such that pre-inocula were established to ODs of 0.500 and 1.500 at 600 nm. A volume of 0.5 mL of each of these pre-inocula was applied to 125-mL flasks, each containing 9.5 mL liquid medium (total of 10 mL culture). These liquid media consisted of LB + 0.5 mM DBT, either with or without 6.65 μM MgSO_4_. To facilitate the measurements and to assure that aliquoting in each time point was not disturbing the culture physiology, various identical 10-mL cultures were set, with the culturing procedure for a single flask being interrupted at each OD measurement time; therefore, seven flasks (6 time points + time ‘zero’) per medium type (4 medium-inoculum combinations) were established, in a total of 28 independent 10-mL cultures, rigorously inoculated in the same way. Controls were the same media with absence of the microorganism. The experiment was performed three times, showing adequate consistency and reproducibility of results.

### Resting-cells experiments

To evaluate DBT consumption/degradation in these assays, the following procedures were performed. From colonies grown in Petri dishes containing LB medium supplemented with DBT (first-step culture), aliquots consisting of a loopful of cells were directly transferred to two 1-L flasks, each containing 500 mL of DBT-supplemented (0.5 mM) LB medium, with and without MgSO_4_. After growth for 48 h, these cultures were centrifuged at 3 Krpm for 5 min for bacterial cells concentration. After complete removal of supernatant, the formed pellet was subjected to three washes, each with 25 mL phosphate buffer (0.1 M; pH 7.0), with the final cell mass being placed into 1-L flasks containing 500 mL of only the phosphate buffer and thoroughly mixed. The first 1-mL aliquot corresponding to time ‘zero’ was withdrawn immediately after the addition to phosphate buffer and the cell suspension was set under constant shaking at 150 rpm and 30°C. Further 1-mL aliquots were hourly withdrawn for 10 hours, for hexane-based extraction (1 : 1) of DBT (see below). The average dry weight of the resting cells that were incubated in phosphate buffer was assessed right after the final centrifugation step. Five culture samples grown and processed in the same manner just described were oven-dried at 60°C for 24 h and weighed; the average weight of cell masses at this stage ranged from 1.6 – 1.7 g.

The experiment was performed three times, showing adequate consistency and reproducibility of results.

### Substrate consumption and product formation during growth in DBT

DBT consumption and 2-hydroxybiphenyl (HBP) formation were determined in culture prior to, during and after growth in LB medium containing DBT, as well as in the resting-cells assays. Both compounds were extracted with hexane at 1 : 1 ratio, by 30 min shaking at 200 rpm and 30 min sonication (MaxiClean 1600). After the mixture was left resting for an appropriate time to separate the phases, the supernatant (organic phase) was analyzed by high-performance liquid chromatography, using a Shimadzu® HPLC system (Kyoto, Japan) device equipped with an UV detector (SPD-20A) set at a wavelength of 248 nm. Compounds detection and quantification were achieved by using a reversed-phase column (Gemini C18 150 × 4.60 mm, 5 μm) from Phenomenex® (Torrance, CA, USA). The analytical conditions consisted of a mobile phase, using an isocratic system composed of 60% acetonitrile and 40% water [26], which was sonicated for 30 min. The compounds identification was based on matching retention times for the samples components to the retention time for standard samples, assessed under the same working conditions. Media or phosphate buffer containing DBT without inoculated cells, and at time ‘zero’ of incubation (at inoculation), were also subjected to extraction for these components, to serve as controls for the sensitivity of the quantification method; practically 100% of the DBT applied was recovered by the evaluation technique after incubation time in cell-free media/buffer, confirming the high sensitivity and precision of measurements.

## Abbreviations

SO_2_^−^: Sulphur dioxide
DBT: Dibenzothiophene
rDNA: Gene sequence for ribosomal rRNA subunit
HBP: 2-hydroxybiphenyl
DBTO: DBT sulfoxide
DBTO_2_: DBT sulfone
HBPS: 2-hydroxybiphenyl-2-sulfinate
LB: Luria-Bertani culture medium
TY: Triptone-Yeast Agar medium
UV: Ultraviolet light
PCR: Polymerase chain reaction
OD: Optical density (in culture)
MgSO_4_: Magnesium sulphate
HPLC: High performance liquid chromatography
DszB: DBTS-monooxygenase
DszC: 2-Hidroxybiphenyl-2-sulfinato desulfinase
